# Analytical Optimal Load Calculation of RF Energy Rectifiers Based on a Simplified Rectifying Model †

**DOI:** 10.3390/s21238038

**Published:** 2021-12-01

**Authors:** Lichen Yao, Guido Dolmans, Jac Romme

**Affiliations:** 1 Electronics System, Department of Electrical Engineering, Eindhoven University of Technology, 5600 MB Eindhoven, The Netherlands; guido.dolmans@imec.nl; 2 Holst-Centre, IMEC-NL, 5656 AE Eindhoven, The Netherlands; jac.romme@imec.nl

**Keywords:** WPT, RF, rectifier, load resistance, analytical, closed-form, half wave, voltage multiplier

## Abstract

Wireless power transfer (WPT) is an essential enabler for novel sensor networks such as the wireless powered communication network (WPCN). The efficiency of an energy rectifier is dependent on both input power and loading condition. In this work, to maximize the rectifier efficiency, we present a low-complexity numerical method based on an analytical rectifier model to calculate the optimal load for different rectifier topologies, including half-wave and voltage-multipliers, without needing time-consuming simulations. The method is based on a simplified analytical rectifier model based on the diode equivalent circuit including parasitic parameters. Furthermore, by using Lambert-W function and the perturbation method, closed-form solutions are given for low-input power cases. The method is validated by means of both simulations and measurements. Extensive transient simulation results using different diodes (Skyworks SMS7630 and Avago HSMS285x) and frequency bands (400 MHz, 900 MHz, and 2.4 GHz) are provided for validation of the method. A 400 MHz 1- and 2-stage voltage multiplier are designed and fabricated, and measurements are conducted. Different input signals are used when validating the proposed methods, including the single sinewave signal and the multisine signal. The proposed numerical method shows excellent accuracy with both signal types, as long as the output voltage ripple is sufficiently low.

## 1. Introduction

Wireless power transfer (WPT) is an emerging technology that removes the traditional charging cables. Due to its convenience, WPT can be found or foreseen in many applications such as electric vehicles, consumer electronics, and new communication networks [1,2]. Specifically, far-field WPT based on RF signals can deliver wireless power over a long distance up to kilometers, which enables new communication and sensing networks in the IoT domain such as wirelessly powered communication network (WPCN). These networks consist of low-power sensor nodes whose power is provided by either dedicated RF sources or ambient RF energy, which prolongs the sensors’ lifetime and reduces the maintenance cost [3].

The receiving side of an RF WPT system is called an energy rectifier. The rectifier converts the RF signal into a DC voltage that either directly powers electronics or is stored in storage units such as batteries or super-capacitors. It often consists of an antenna that captures the wireless signal; a matching and rectifying network for RF-to-DC conversion; and a power management unit (PMU), which is the rectifier’s load. The power conversion efficiency (PCE) of a rectifier has been shown to depend on both received RF power and its load; thus, the optimal load for a rectifier needs to be understood [4]. Additionally, novel excitation waveforms such as multisine waveform featuring high peak-to-average-power ratio (PAPR) and multiple frequency components [5] have been proposed to boost the rectification efficiency. To analyze the optimal load with general excitation, transient simulations are often conducted [6], which are time-consuming and computationally intensive. Harmonic balance (HB) is another option, but its complexity poorly scales with the number of frequency components in the excitation waveform so that it soon becomes impractical [7].

Instead of numerical solvers, many efforts were put into the analytical modeling of rectification. The works [8,9,10] used the time-domain method to analytically analyze the shunt-diode rectifier. Afterwards, ref [11] the model was extended with class-f harmonic termination. In [12,13,14], Bessel functions are used to separate DC component and the first harmonic of the diode voltage and the optimal load using the developed model is calculated. A limitation to the aforementioned works is that they all assume the applied excitation to be a sine wave. Works in [15,16] extensively analyzed the incurred losses in the complete rectification chain and pointed out the optimal load resistance for the overall efficiency in the low input power range is equal to the diode junction resistance and series resistance combined. The junction resistance, however, depends on the junction bias voltage, which in turn depends on the load; thus, additional steps are needed to calculate or measure this quantity.

There have also been works focusing on developing analytical rectification models for general multisine signals. In [17], a simplified analytical model was developed to mathematically prove the efficiency gain of the multisine excitation. Later, this model was used in [18,19] to optimize the transmission waveform with frequency-selective fading channels because of the tractability of this rectifier model and its ability to capture the non-linearity of the rectifier circuitry. For the same reason, this model was also used in system performance analysis and optimization of WPCNs and shows superior accuracy to the conventional linear rectifier model in [20,21,22,23]. Despite the successful applications of this model, the key assumptions in [17,18] when developing it are the ideal diode and the half-wave rectifier topology.

In our previous work [24], the model with diode parasitics in the simplest half-wave rectifier was discussed; then, the model was extended for the voltage-doubler. We also showed the low-complexity method to derive the optimal load. The method works with general multisine input signals, provided that the output voltage ripple is small enough. In the current work, we further extend the model to generic N-stage voltage-multipliers. More extensive transient simulations are conducted to validate the result. Two different Schottky diodes are considered in the simulation, and three different frequency bands: 400 MHz, 900 MHz, and 2.4 GHz, which are simulated as well to investigate the impact of frequency. Finally, rectifier prototypes are designed and fabricated and a measurement campaign is conducted to provide experimental data to further support the results.

The paper is organized as follows: Section 2 introduces the simplified analytical rectifier model for both half-wave and N-stage voltage multiplier with a realistic diode equivalent model; Section 3 describes the calculation of the optimal load and its closed-form asymptotic solutions; and numerical and experimental validations are discussed in Section 4, including the simulation setup, PCB design considerations, measurement setup, results, interpretation, and discussion. Finally, Section 5 summarizes the paper and discusses the implications of applications.

## 2. Analytical Rectification Model

In this section, we will summarize the rectification model for the half-wave topology and analyze the effects of diode parasitic parameters. Then, we will extend the model to a generic N-stage voltage multiplier.

### 2.1. Half-Wave Rectification Model

**Figure 1:**
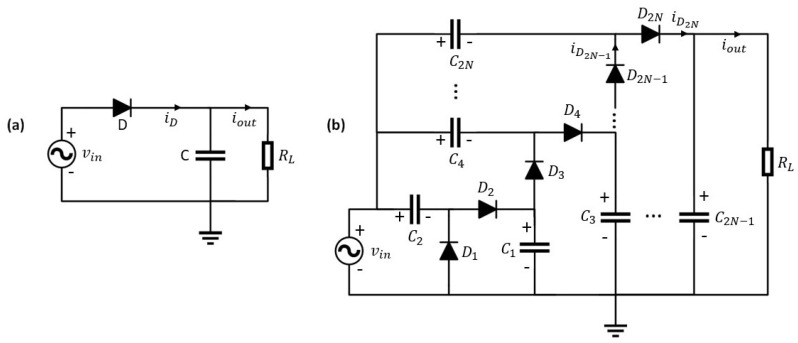
Schematic of (**a**) a half-wave rectifier and (**b**) an N-stage voltage-multiplier.

**Figure 2:**
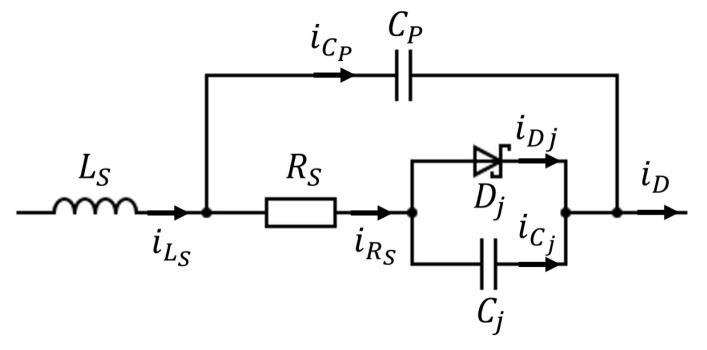
Diode equivalent circuit [25].

The schematic of a half-wave rectifier is shown in Figure 1a. The diode is modeled by the equivalent circuit shown in Figure 2, where there is the ideal diode junction $D_{j}$, junction capacitance $C_{j}$, series resistance $R_{S}$, parallel capacitance $C_{P}$, and series inductance $L_{S}$. Assume a multisine input voltage to the rectifier circuit being:

(1) $$v_{in}\left(t\right)=\sum_{n=0}^{N_{f}-1}V_{A}\cos\left(2\pi f_{n}t+\phi_{n}\right)$$

where $N_{f}$ is the number of sub-carriers or tones, $V_{A}$ is the amplitude of each tone, $f_{n}$ and $\phi_{n}$ are the frequency and phase of the *n*-th tone, respectively. The tones are assumed to follow a uniform frequency grid, such that $f_{n}=f_{0}+n\Delta_{f}$, with $\Delta_{f}$ being the frequency separation. As a result, $v_{in}\left(t\right)$ is a periodic signal with period $T=1/\Delta_{f}$ when $N_{f}>1$. The CW signal can be viewed as a special case with $N_{f}=1$ and a period of $T=1/f_{0}$.

According to the Kirchhoff’s voltage and current law, and Figure 1a and Figure 2, we have the following relationships:

(2) $$\begin{array}{cc} C\frac{dv_{out}\left(t\right)}{dt}+i_{out}\left(t\right) & =C_{P}\frac{dv_{C_{P}}\left(t\right)}{dt}+i_{D_{j}}\left(t\right)+C_{j}\frac{dv_{C_{j}}\left(t\right)}{dt} \end{array}$$

(3) $$\begin{array}{cc} i_{D_{j}}\left(t\right) & =i_{s}\left(e^{\alpha v_{D_{j}}\left(t\right)}-1\right) \end{array}$$

(4) $$\begin{array}{cc} v_{D_{j}}\left(t\right) & =v_{in}\left(t\right)-v_{L_{S}}\left(t\right)-v_{R_{S}}\left(t\right)-v_{out}\left(t\right) \end{array}$$

(5) $$\begin{array}{cc} i_{R_{S}}\left(t\right) & =i_{D}\left(t\right)-C_{P}\frac{dv_{C_{P}}\left(t\right)}{dt} \end{array}$$

where $i_{s}$ is the diode saturation current, and $\alpha =1/(nv_{t})$ with *n* and $v_{t}$ being ideality factor and thermal voltage, respectively. Equation (3) is the Shockley equation of the diode junction. Because we are interested in the DC output voltage rather than its transient, we average both sides of Equation (2) over a signal period after the system reaches steady state:

(6) $$i_{dc}=E\left\{i_{D_{j}}\left(t\right)\right\}=\frac{i_{s}}{T}\int_{0}^{T}e^{\alpha v_{D_{j}}\left(t\right)}dt-i_{s}$$

where $i_{dc}$ is the DC component of output current $i_{out}$, E{.} denotes time averaging, and the juntion current $i_{D_{j}}\left(t\right)$ is substituted by Equation (3). Note that during the time averaging, the current terms related to capacitors vanishe. This is because in the steady state, the amount of electronic charges on a capacitor remains the same in the beginning and the end of a period. As a result, the average current has to be zero.

Next, we assume the output capacitor *C* in Figure 1a is sufficiently large, such that the output voltage ripple is negligible. Hence, the output voltage over the load is effectively a DC signal, such that we can write $i_{out}\left(t\right)\approx i_{dc}$ and $v_{out}\left(t\right)\approx i_{dc}R_{L}$. This is a reasonable assumption since a steady state voltage source is essential for the proper functionality of the circuitry behind the rectifier. Following this assumption, we can further approximate the current through diode $i_{D}\left(t\right)=C\frac{dv_{out}\left(t\right)}{dt}+i_{out}\left(t\right)\approx i_{dc}$. Substituting these approximations in Equation (4), we obtain

(7) $$v_{D_{j}}\left(t\right)\approx v_{in}\left(t\right)-i_{R_{S}}\left(t\right)R_{S}-i_{dc}R_{L}$$

The series inductance term is dropped because $L_{S}$ is typically very small so naturally $v_{L_{S}}\left(t\right)=L_{s}\frac{di_{D}\left(t\right)}{dt}\approx 0$. Similarly, the parallel capacitance term in Equation (5) is also dropped due to small $C_{P}$ value. As a result, Equation (5) can be rewritten by $i_{R_{S}}\left(t\right)\approx i_{dc}$, and Equation (7) is now:

(8) $$v_{D_{j}}\left(t\right)\approx v_{in}\left(t\right)-i_{dc}\left(R_{S}+R_{L}\right)$$

Substitute it back to Equation (6):

(9) $$i_{dc}=e^{-\alpha i_{dc}\left(R_{S}+R_{L}\right)}\frac{i_{s}}{T}\int_{0}^{T}e^{\alpha v_{in}\left(t\right)}dt-i_{s}$$

Note now that $i_{dc}$ is still on both sides of the equation. Move $i_{s}$ to the left hand side and multiply $\alpha R_{h}e^{\alpha \left(i_{dc}+i_{s}\right)R_{h}}$ to both sides:

(10) $$\alpha \left(i_{dc}+i_{s}\right)R_{h}e^{\alpha \left(i_{dc}+i_{s}\right)R_{h}}=\alpha R_{h}e^{\alpha i_{s}R_{h}}\frac{i_{s}}{T}\int_{0}^{T}e^{\alpha v_{in}\left(t\right)}dt$$

where $R_{h}=R_{S}+R_{L}$. Equation (10) can be solved for $i_{dc}$ by using the principle branch of the Lambert W-function [26]:

(11) $$i_{dc}\left(v_{in},R_{L}\right)=-i_{s}+\frac{1}{\alpha R_{h}}W\left(\alpha R_{h}\left(i_{s}+z_{dc}\right)e^{\alpha i_{s}R_{h}}\right)$$

where $z_{dc}=\frac{i_{s}}{T}\int_{0}^{T}e^{\alpha v_{in}\left(t\right)}dt-i_{s}$, which is a monotonic function with the amplitude of input voltage, and W(x) is the Lambert W-function whose value is the solution of *w* to the equation $we^{w}=x$. The Lambert W-function does not have an explicit formula but can be evaluated by simple numerical methods described in [26].

### 2.2. N-Stage Voltage-Multiplier Rectification Model

In this section, we will generalize the analytical half-wave rectification model developed in the previous section to *N*-stage voltage-multiplier. Figure 1b shows the schematic of a *N*-stage voltage-multiplier. A voltage-multiplier is often used to boost the output DC voltage by cascading voltage-doublers. The capacitors $C_{n}$ with even *n* are used to provide DC offset to each stage so the output voltage is stepped up gradually. Assume all diodes used in Figure 1b are the same. According to the Kirchhoff’s current law, for the upper diode of the last stage $D_{2N}$:

(12) $$C_{2N-1}\frac{dv_{out}\left(t\right)}{dt}+i_{out}\left(t\right)=C_{2N}\frac{dv_{C_{2N}}\left(t\right)}{dt}+C_{P_{2N}}\frac{dv_{C_{P(2N)}}\left(t\right)}{dt}+i_{D_{j(2N)}}\left(t\right)+C_{j(2N)}\frac{dv_{C_{j(2N)}}\left(t\right)}{dt}$$

where $C_{P(2N)}$ and $D_{j(2N)}$ denote the parallel capacitance and junction of the 2N-th diode. By time-averaging the above equation in the steady state like we did with Equation (2), we get:

(13) $$i_{dc}=E\left\{i_{D_{j(2N)}}\left(t\right)\right\}=\frac{i_{s}}{T}\int_{0}^{T}e^{\alpha v_{D_{j(2N)}}\left(t\right)}dt-i_{s}$$

According to the Kirchhoff’s voltage law for the diode $D_{2N}$, the junction voltage is:

(14) $$v_{D_{j(2N)}}\left(t\right)=v_{in}\left(t\right)-v_{C_{2N}}\left(t\right)-v_{L_{S(2N)}}\left(t\right)-v_{R_{S(2N)}}\left(t\right)-v_{out}\left(t\right)$$

where $L_{S(2N)}$ and $R_{S(2N)}$ are series inductance and series resistance of the 2N-th diode. The same treatment with the series inductance and resistance can be done as when analyzing the half-wave rectifier, to approximate $v_{L_{S(2N)}}\approx 0$ and $v_{R_{S(2N)}}\approx i_{dc}R_{S}$. To ensure a small output ripple, all capacitors in a voltage-multiplier need to be large enough so the time constant is larger than the signal period. This means the capacitors can be considered short-circuits at high frequency so that their voltage drop has only DC component [27]. At DC, the capacitors are open circuit and the input is shorted because the input voltage does not have DC component. As a result, the voltage drop across $C_{2N}$ is:

(15) $$v_{C_{2N}}\left(t\right)=-\frac{2N-1}{2N}i_{dc}R_{L}$$

which equals to the voltage drop across the first 2N−1 cascaded diodes. Using it in Equations (13) and (14), we get:

(16) $$i_{dc}=e^{\alpha i_{dc}\left(R_{S}+\frac{R_{L}}{2N}\right)}\frac{i_{s}}{T}\int_{0}^{T}e^{\alpha v_{in}\left(t\right)}dt-i_{s}$$

Again, solve it for $i_{dc}$ using the principle branch of the Lambert W-function:

(17) $$i_{dc}\left(v_{in},R_{L}\right)=-i_{s}+\frac{1}{\alpha R}W\left(\alpha R\left(i_{s}+z_{dc}\right)e^{\alpha i_{s}R}\right)$$

where $R=R_{S}+\frac{R_{L}}{2N}$ and *N* is the number of stages of a voltage-multiplier. Given the similarity between Equation (11) for half-wave and Equation (17) for voltage-multiplier, the half-wave model can be viewed as a special case of the multiplier model with number of stages N=0.5.

## 3. Calculation of Optimal Load Resistance

### 3.1. Problem Formulation

We have so far developed the output DC current in the last chapter. By definition, the output DC power $P_{dc}$ is:

(18) $$P_{dc}=i_{dc}^{2}R_{L}$$

The optimal load that maximizes $P_{dc}$ can be found by numerically evaluating Equation (18) based on Equation (17) with a scanned $R_{L}$. This solution is called numerical solution of the analytical model.

To find the closed-form solution to the optimal load, the first derivative of $P_{dc}$ needs to be formulated. We first write the $i_{dc}$’s first derivative with respect to the load using (17):

(19) $$\frac{\partial i_{dc}}{\partial R_{L}}=-\frac{1}{2\alpha NR^{2}}W\left(E\right)+\frac{1}{\alpha R}\frac{\partial W(E)}{\partial R_{L}}$$

where $E=\alpha R\left(i_{s}+z_{dc}\right)e^{\alpha i_{s}R}$. To simplify the notation, we omit the dependency of both $i_{dc}$ and $P_{dc}$ on $v_{in}$ and $R_{L}$ in equations from here on. The derivative of $P_{dc}$ with respect to the load resistance:

(20) $$\frac{\partial P_{dc}}{\partial R_{L}}=i_{dc}\left(i_{dc}+2R_{L}\frac{\partial i_{dc}}{\partial R_{L}}\right)$$

Use Equations (17) and (19) in (20) we further have:

(21) $$\frac{\partial P_{dc}}{\partial R_{L}}=i_{dc}\left(-i_{s}+\frac{NR-R_{L}}{\alpha NR^{2}}W\left(E\right)+\frac{2R_{L}}{\alpha R}\frac{\partial W(E)}{\partial R_{L}}\right)≜i_{dc}I_{0}$$

Since $i_{dc}$ is by definition a non-negative number, solving for $I_{0}=0$ is equivalent to solving $\frac{\partial P_{dc}}{\partial R_{L}}=0$. However, finding the closed-form solution can be a challenge due to the lack of explicit formula of the W-function.

### 3.2. Closed-Form Approximations for Low Input Power

The W-function can be approximated in closed-form under some assumptions. By definition, the value of the W-function in Equation (21) is the solution of the equation:

(22) $$W\left(E\right)e^{W(E)}=\alpha i_{s}Re^{\alpha i_{s}R}+\alpha z_{dc}Re^{\alpha i_{s}R}$$

An easy solution would be obtained if the second term on the right hand side was absent, which is $W\left(E\right)=\alpha i_{s}R$. This situation is similar to solving a nonlinear ordinary differential equation (ODE). When the ODE is constructed in a way that there is a simple part added by a complex nonlinear term, often the perturbation method can be applied if the nonlinear term is small [28]. Here, we apply the perturbation method to solve Equation (22) under the condition that $z_{dc}$ is small compared with $i_{s}$. Because $z_{dc}$ is a monotonic function of input voltage amplitude, the condition is equivalent to a small input power.

The exact solution W(E) is obviously a function of $z_{dc}$; thus, a power series about $z_{dc}$ exists that approximates W(E):

(23) $$W\left(E\right)\approx W_{a}^{\left(K\right)}\left(E\right)=\sum_{k=0}^{K}z_{dc}^{k}W_{k}$$

where $W_{a}^{\left(K\right)}\left(E\right)$ is the approximation to W(E) with order *K* and the coefficients $W_{k}$, ∀k=0,1,…,K are the generating solutions. Naturally, the smaller $z_{dc}$ is, the less order *K* is needed before the approximation converges. After substituting (23) into (22) and taking logarithm on both sides, we obtain

(24) $$\ln\left(\sum_{k=0}^{K}z_{dc}^{k}W_{k}\right)+\sum_{k=0}^{K}z_{dc}^{k}W_{k}=\ln\left(\alpha (i_{s}+z_{dc})R\right)+\alpha i_{s}R$$

Taking the derivative of this equation from 0 to *K* times and equating $z_{dc}$ to zero each time gives us K+1 generating equations. We list them with K=2 here:

(25) $$\begin{array}{cc} \; & \ln\left(W_{0}\right)+W_{0}=\ln\left(\alpha i_{s}R\right)+\alpha i_{s}R \end{array}$$

(26) $$\begin{array}{cc} \; & \frac{1}{W_{0}}W_{1}+W_{1}=\frac{1}{i_{s}} \end{array}$$

(27) $$\begin{array}{cc} \; & -{\left(\frac{W_{1}}{W_{0}}\right)}^{2}+2\frac{W_{2}}{W_{0}}+2W_{2}=-\frac{1}{i_{s}^{2}} \end{array}$$

Then, it is straightforward to get the generating solutions:

(28) $$\begin{array}{c} W_{0}\;=\;\alpha i_{s}R,W_{1}\;=\;\frac{\alpha R}{1\;+\;\alpha i_{s}R},W_{2}\;=\;-\frac{\alpha^{2}R^{2}\left(2\;+\;\alpha i_{s}R\right)}{2{\left(1\;+\;\alpha i_{s}R\right)}^{3}} \end{array}$$

Using the first two generating solutions and (23), the W(E) is approximated in order K=1 by:

(29) $$W_{a}^{\left(1\right)}\left(E\right)=\alpha i_{s}R+\frac{\alpha Rz_{dc}}{1+\alpha i_{s}R}$$

Substitute this into $I_{0}=0$ and solve for $R_{L}$, the optimal resistance based on the 1st order approximation is:

(30) $$R_{L,1}^{*}=2N\left(R_{S}+\frac{1}{\alpha i_{s}}\right)$$

This solution is then the closed-form approximation with the first-order truncation for extremely low input power. Note that what is inside the bracket is the diode’s resistance at low power [29], which suggests the load should match the resistance of all diodes in series to obtain maximum output power.

Furthermore, using all three generating solutions in (28), the W(E) is approximated in order K=2 by the following:

(31) $$\begin{array}{c} W_{a}^{\left(2\right)}\left(E\right)=W_{a}^{\left(1\right)}\left(E\right)-\frac{\alpha^{2}R^{2}\left(2+\alpha i_{s}R\right)z_{dc}^{2}}{2{\left(1+\alpha i_{s}R\right)}^{3}} \end{array}$$

This approximation of higher order is accurate over a wider $z_{dc}$ range than the 1st order approximation in (29). By substituting this into $I_{0}=0$, multiplying a positive term $\alpha {\left(\alpha^{-1}+i_{s}R\right)}^{4}/\left(i_{s}^{2}z_{dc}\right)$ to both sides of the equation, and simplifying, we get a cubic equation about *R*:

(32) $$\begin{array}{c} \mu R^{3}+\frac{\mu }{\alpha i_{s}}R^{2}-\frac{\mu +5z_{dc}/2}{\alpha^{2}i_{s}^{2}}R+\frac{1}{\alpha^{3}i_{s}^{2}}=0 \end{array}$$

where $\mu =z_{dc}/2-i_{s}$. Since only positive multipliers are used during the derivation of (32), the Equation (32) is equivalent to $\frac{\partial P_{dc}}{\partial R_{L}}=0$. During the simplification of the above equation, we used approximation $R\approx R_{L}/2N$ in order to simplify the derivation. This is supported by the fact that the diode series resistance $R_{S}$ is normally no more than a few tens of Ohm while the optimal load is typically in the order of kilo-Ohm. As the optimal load decreases to lower magnitudes with higher input power, the approximation (31) will become inaccurate, as we will show in Section 4 for validation results. The solution to the above cubic function is found by using Cardano’s general cubic formula. The roots of a cubic equation $ax^{3}+bx^{2}+cx+d=0$ are given by:

(33) $$\begin{array}{c} x_{k}=-\frac{1}{3a}\left(b+\xi^{k}B+\frac{\Delta_{0}}{\xi^{k}B}\right),\;\;k\in \left\{0,1,2\right\} \end{array}$$

where $x_{k}$ is the *k*-th root, $B=\sqrt[{3}]{\frac{\Delta_{1}\pm \sqrt{\Delta_{1}^{2}-4\Delta_{0}^{3}}}{2}}$, $\Delta_{0}=b^{2}-3ac$, $\Delta_{1}=2b^{3}-9abc+27a^{2}d$, $\xi =\frac{-1+\sqrt{-3}}{2}$. The choice of plus or minus in *B* is arbitrary as long as it does not lead to B=0. We then choose the smallest positive real root out of the three, i.e.,

(34) $$\begin{array}{c} R_{L,2}^{*}=\min_{\forall k\in \Theta }\left(2Nx_{k}\right) \end{array}$$

where $\Theta =\{k\in \left\{0,1,2\right\}|x_{k}\;\mathrm{is}\;\mathrm{real}\;\mathrm{and}\;\mathrm{positive}\}$.

**Theorem** **1.** *The smallest positive root of (32) is the optimal load resistance that maximizes $P_{dc}$ when $z_{dc}<2i_{s}$.*

**Proof** **of** **Theorem** **1.** Denote the left hand side of (32) by $C_{I_{0}}$. $C_{I_{0}}$ has a positive y-intercept $\frac{1}{\alpha^{3}i_{s}^{2}}$, so its positive before *R* increases to its minimum positive root and becomes negative after that. $C_{I_{0}}$ has the same polarity as partial derivative $\frac{\partial P_{dc}}{\partial R_{L}}$ because there is only a positive term multiplied to $I_{0}$, which means the first positive root is a local maximizer of $P_{dc}$. It can be easily proven that $C_{I_{0}}$ either monotonically decreases or increases first then decreases when $z_{dc}<i_{s}$ by inspecting $C_{I_{0}}$’s derivative. This means $C_{I_{0}}$ always has a single positive root when $z_{dc}\leq 2i_{s}$; thus, the local maximizer is also a global maximizer. When $z_{dc}\geq 2i_{s}$, there may be more than one positive root, but the small $z_{dc}$ assumption is violated so the perturbation approximation is inaccurate anyway.    □

## 4. Validation and Discussions

The validation consists of two parts: simulation and measurement, whose details will be explained in this section. The results and insights obtained from the validation will also be discussed.

### 4.1. Simulation Setup and Results

To verify the accuracy of the proposed methods, a set of transient simulations are carried out with MATLAB Simscape Electrical [30] by sweeping the load $R_{L}$. The load sweep starts from 10 Ω to several tens of kΩ. Two low-barrier Schottky diodes Skyworks SMS7630 and Avago HSMS285x are used for comparison. Key parameters of the two considered types of diode are taken from their data sheets and summarized in Table 1. In Simscape, the junction capacitance $C_{J}$ is modeled as a voltage dependent parameter calculated based on zero-bias junction capacitance $C_{J0}$, junction potential $V_{J}$, and grading coefficient *M*. All capacitors in Figure 1 are set to 500 pF. Besides, the simulation is carried out in three different frequency bands: 400 MHz, 900 MHz, and 2.4 GHz, due to their availability of license-free bands.

**Figure 3:**
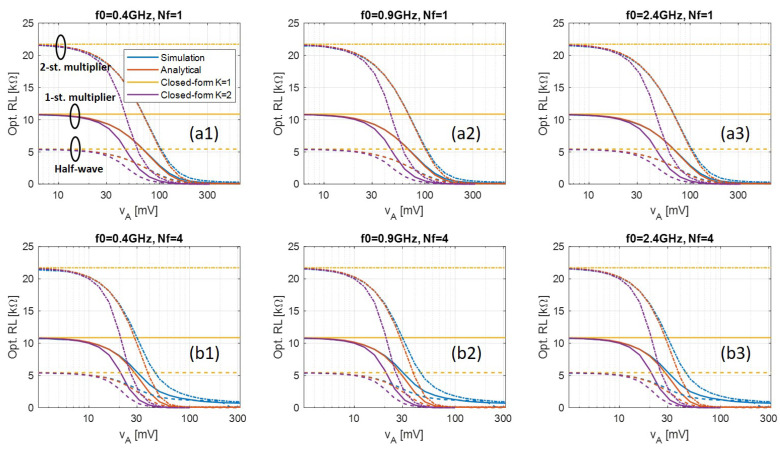
Optimal load calculated by transient simulation (blue), numerical solution of the analytical model (red), and closed-form solutions (yellow and purple), when (**a**) $N_{f}=1$, and (**b**) $N_{f}=4$ with $\Delta_{f}=1$ MHz, with half-wave, 1-, and 2-stage voltage-multipliers using Skyworks SMS7630. The results under different frequency bands (**a1**,**b1**) 400 MHz, (**a2**,**b2**) 900 MHz, and (**a3**,**b3**) 2.4 GHz are shown, too.

Figure 3(a1) shows the calculated optimal load of three different rectifier topologies, i.e., half-wave, 1-, and 2-stage voltage-multipliers with Skyworks SMS7630 diode at 400 MHz frequency band. It can be seen that the numerical solution to the analytical model (red) has good accuracy compared with simulated results (blue) for all topologies. This proves that our proposed simplified analytical model is sufficiently accurate and that no numerical simulation is needed. Besides, the optimal load of 1- and 2-stages voltage-multipliers are roughly 2 and 4 times larger than that of the half-wave rectifier, which corresponds to 2 and 4 times more series diodes from the load’s view point.

Figure 3(a1) also shows the closed-form solutions with truncation order K=1 (yellow) and K=2 (purple). When K=1, the closed-form result is accurate only when input power is extremely low and is an upper bound of optimal load. This is helpful when determining the specification of an adaptive optimal load system. For K=2, the valid input power region is wider until approximately 30 mV of input voltage amplitude, after which it becomes inaccurate. This completely closed-form solution is helpful in very low power applications due to its extremely low computational complexity.

Figure 3(a2,a3) also shows results at 900 MHz and 2.4 GHz bands. No clear difference can be observed when frequency is increased to 2.4 GHz. This is because the Skyworks diode has very small parasitic parameters, see Table 1, which means the reactance part of the diode impedance remains negligible within the frequency spectrum that we considered.

Figure 3(b1–b3) shows the calculated optimal load at three different frequency bands, with multisine signals consisting of four subcarriers that are separated by 1 MHz. The numerical solution to the analytical model (red) still shows high accuracy in low input power region, while larger discrepancy is observed as input amplitude increases than when $N_{f}=1$. This is because a much larger signal period and thus a much larger ripple is created by the multisine signal. As a result, to minimize the output ripple, an output R-C section with a much larger time-constant is needed than when single-sinusoid signal is used. In the simulation, the output capacitor is 500 pF all the time, so when the load decreases, it will come to a point when the output ripple becomes significant, which is also when the low ripple assumption of the rectification model fails. In practice, this can be avoided by using a large enough capacitor in the output R-C section based on the applied signal.

**Figure 4:**
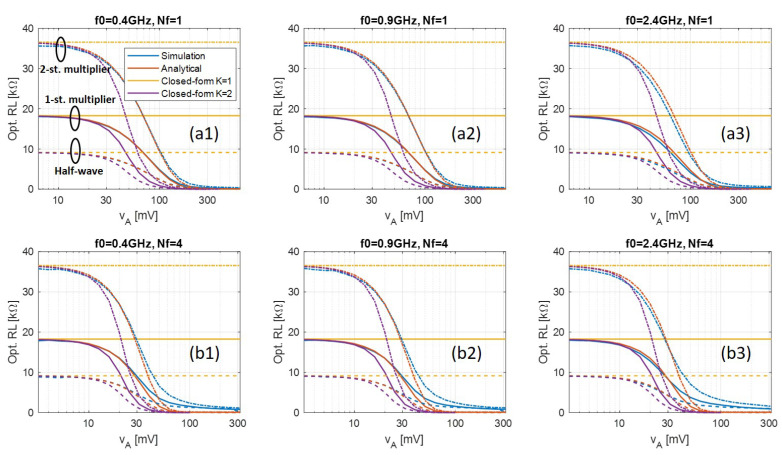
Optimal load calculated by transient simulation (blue), numerical solution of the analytical model (red), and closed-form solutions (yellow and purple) when (**a**) $N_{f}=1$, and (**b**) $N_{f}=4$ with $\Delta_{f}=1$ MHz, simulated with half-wave, 1-, and 2-stages voltage-multipliers using Avago HSMS285x. The results under different frequency bands (**a1**,**b1**) 400 MHz, (**a2**,**b2**) 900 MHz, and (**a3**,**b3**) 2.4 GHz are shown, too.

Figure 4 shows the optimal load calculated by the proposed methods and simulation when using Avago HSMS285x. Similarly to the results with the Skyworks diode, the proposed analytical solution (red) shows very good accuracy compared with simulated results (blue). However, at 2.4 GHz, a slightly larger discrepancy can be observed between the analytical and the simulated results, due to the fact that the Avago diode has considerably larger parasitic capacitance and inductance as can be seen from Table 1. This means at higher frequency, the effect of parasitics becomes more significant while the simplified model neglects it. Nevertheless, the error is still minor within the frequency range that we consider.

Another observation is that the optimal load with the Avago diode is almost twice as large as the Skywork’s. This can be interpreted from the order 1 closed-form solution. The saturation current $i_{s}$ of the Avago diode is almost half of the Skyworks one. According to (30), this corresponds to an approximate twice as large optimal load. This observation shows the optimal load of a rectifier is highly dependent on the diode’s parameter.

### 4.2. Measurement Setup

**Figure 5:**
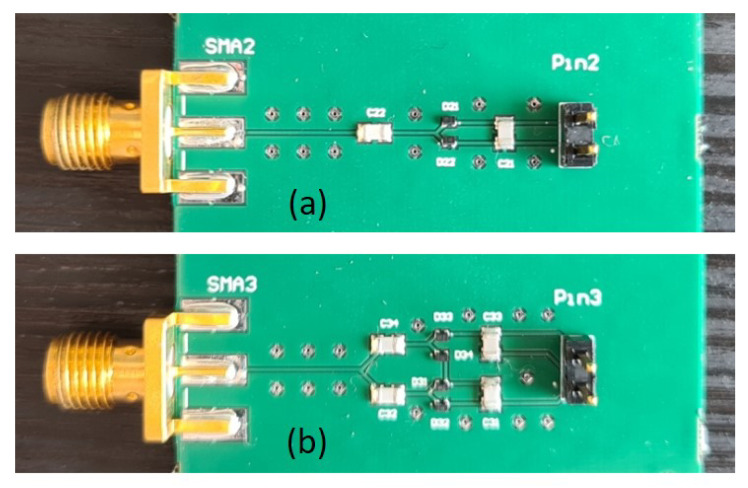
PCB picture of (**a**) one-stage multiplier and (**b**) two-stage multiplier.

To further validate the results, the one-, and two-stage voltage multiplier PCBs are designed in Altium Designer at 400 MHz and fabricated, see Figure 5. Only 400 MHz is chosen for fabrication because of the lack of high-frequency probes for debugging purposes in our lab. An IS400 substrate with dielectric constant $\epsilon_{r}=4.3$ and thickness h=0.119 mm is used. Grounded co-planar waveguide (GCPW) with vias is used as transmission line with track and slot width of 0.225 mm and 0.17 mm to ensure 50 Ω characteristic impedance. An edge-mount SMA connector is used for RF input, and a pin header is used to connect external variable load. All capacitors used on the PCBs are 500 pF. The used Schottky diode is SMS7630-040LF from Skyworks. Measurements have been conducted to obtain experimental data to validate the model presented in Section 2.

**Figure 6:**
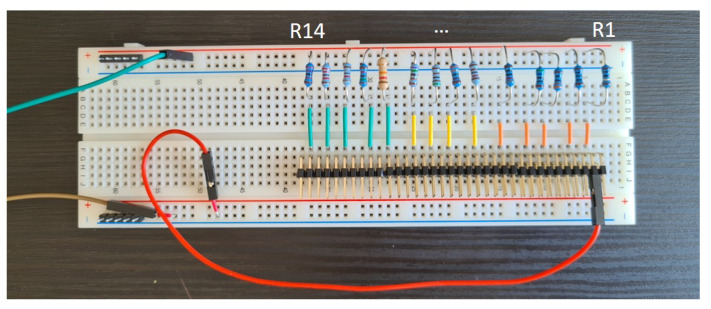
Resistor bank on a bread board.

The variable load is achieved by a resistor bank on a bread board, see Figure 6. In total, there are 14 resistors, and 16 resistance values are used in the measurement. The used resistance values are listed in Table 2.

**Figure 7:**
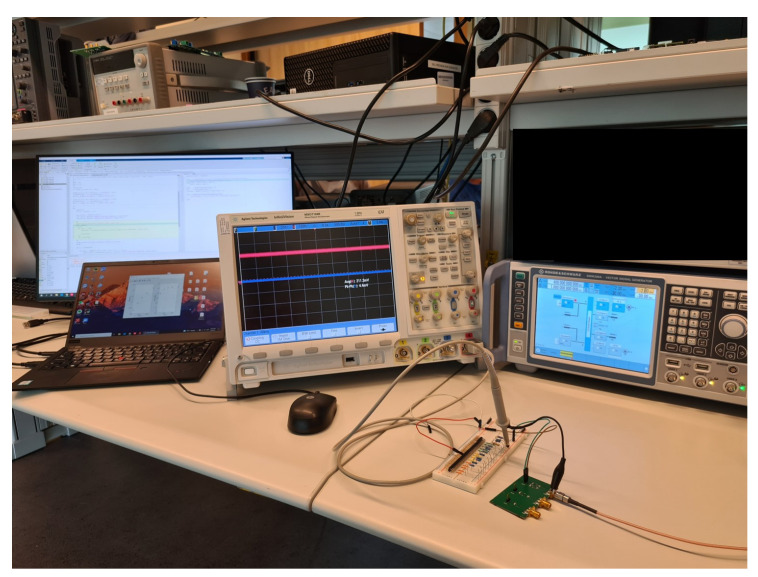
Picture of the measurement setup.

A Rohde & Schwarz SMW200A signal generator is used as RF source to generate the input signal. The RF source is fed to the SMA connector on the PCB using a coaxial RF cable. The average output voltage of the rectifier is measured by a Keysight MSO7104B digital oscilloscope. The acquisition of measured average voltage is controlled by a windows PC through SCPI remote commands via a USB connection. The remote control session is established by MATLAB using Instrument Control Toolbox. The output DC voltage is measured 20 times during each measurement with 0.5 s interval between consecutive acquisitions. The 20 acquired samples are then averaged to obtain a final measurement result. A picture of the measurement setup is shown in Figure 7.

**Figure 8:**
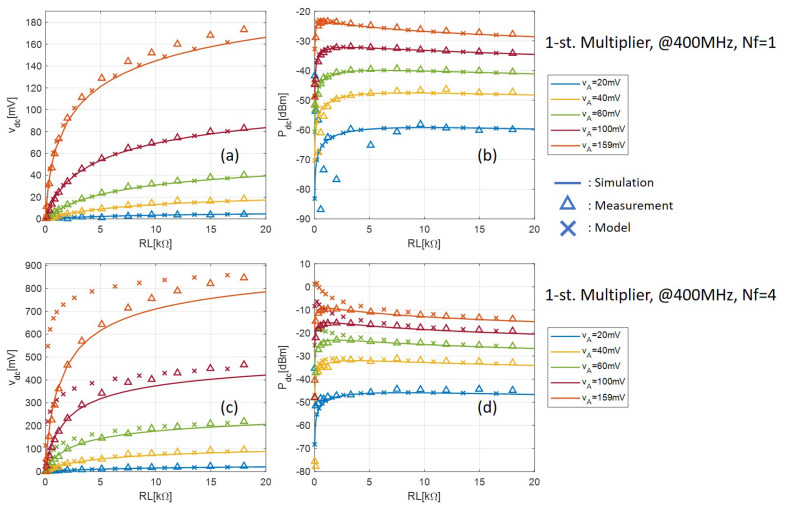
Comparison between measurement, simulation, and analytical model of the one-stage multiplier. Output DC voltage with (**a**) $N_{f}=1$ and (**c**) $N_{f}=4$; output DC power with (**b**) $N_{f}=1$ and (**d**) $N_{f}=4$.

**Figure 9:**
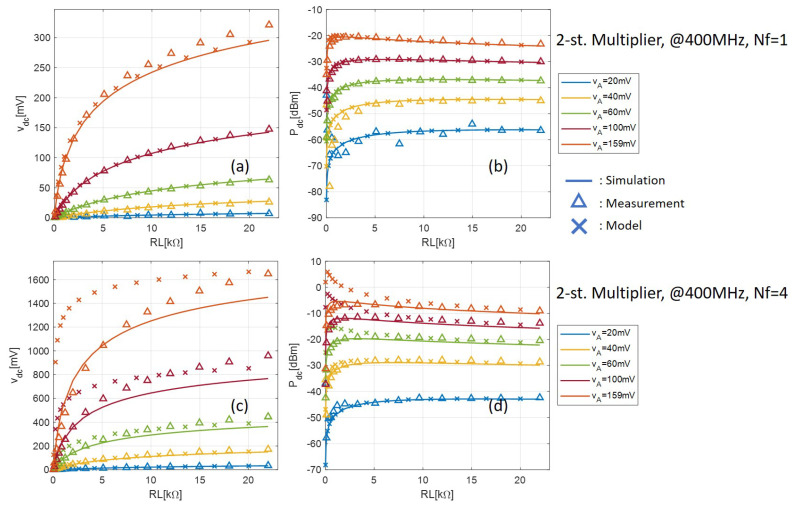
Comparison between measurement, simulation, and analytical model of the two-stage multiplier. Output DC voltage with (**a**) $N_{f}=1$ and (**c**) $N_{f}=4$; output DC power with (**b**) $N_{f}=1$ and (**d**) $N_{f}=4$.

The measured output DC voltage and power with one- and two-stage multipliers are shown in Figure 8 and Figure 9, respectively. The voltage and power are plotted against the load resistance with different tone amplitude $V_{A}$. During measurement, $V_{A}$ is measured at the central pin of the SMA connector on the PCB using a Teledyne LeCroy SDA816Zi serial data analyzer with a ZS1000 active probe with 1 GHz bandwidth. The fluctuation with the measured results with low input $V_{A}$ and especially low load resistance is because of the low output voltage, which is close to the digital oscilloscope’s noise floor. Despite this, the measured and simulated results are consistently in very good agreement.

Figure 8 and Figure 9 also show the output DC voltage and power calculated by the analytical model given by (17) and (18). The accuracy of the analytical model is confirmed with respect to both measured and simulated results when $N_{f}=1$ for all input levels. When $N_{f}=4$, however, the model fails to predict the rectifier output with small loads when the input level is high. This is because high PAPR signals in general (in this case the multisine signal) need an R-C section with a higher time constant than the conventional single-sine signal to eliminate the output voltage ripple, and a negligible ripple is a prerequisite for our simplified model, as we explained in Section 4.1. Indeed from Figure 8 and Figure 9, as the load increases, which leads to a larger time-constant, the model becomes more and more accurate.

**Figure 10:**
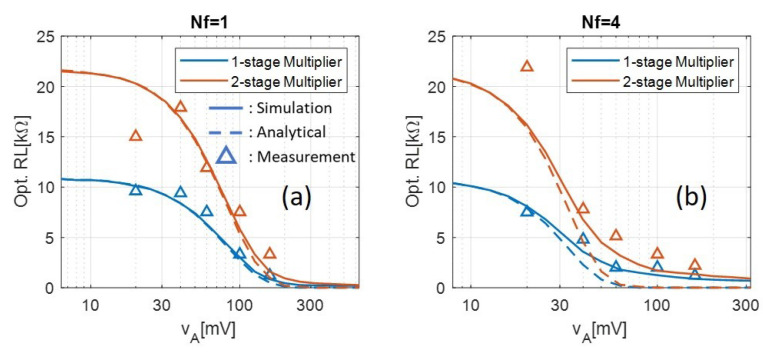
Optimal loads calculated by simulation, numerical solution of the analytical model, and measurement with (**a**) $N_{f}=1$ and (**b**) $N_{f}=4$. Note that the measured results with the lowest input tone amplitude $v_{A}$ tend to be outliers since the rectifier’s output voltage is close to the noise floor of the oscilloscope.

Moreover, the optimal load based on the measured data as a function of $V_{A}$ is shown in Figure 10. The simulated results and the numerical solution of the analytical model are also shown as solid and dashed lines, respectively, for comparison. Note that the resolution of the measured optimal load is limited by the step size of the variable load listed in Table 2. Also note that the optimal load associated with the lowest input level tends to be an outlier since the rectifier’s output voltage is close to the oscilloscope’s noise floor, so more randomness is observed on the left-most measured point in Figure 10a,b. It can be seen that the simulated data is in close agreement with the measured data when $N_{f}=1$ and $N_{f}=4$. The numerical solution of the analytical model also shows great agreement with the measured data except in the high input $V_{A}$ region with four-tone multisine signal, which has already been explained before.

## 5. Conclusions

In this paper, we analyzed simplified analytical rectification models for the half-wave rectifier and the *N*-stage voltage-multiplier. The targeted rectifier topologies are generic, and the models consider the diode as its realistic equivalent circuit. Based on the models, a set of methods that calculate the optimal loading condition for the rectifiers are given, including a low-complexity numerical method, and closed-form approximations for low input power scenarios. The proposed methods are validated by both simulation and measurement.

The simulation results show that the parameters of the diode, namely, saturation current $i_{s}$ and ideality factor *n*, significantly influence the optimal loading condition. The simulation results also show that the carrier frequency does not influence the optimal loading condition with Skyworks SMS7630 diode. The effect of frequency gets larger only when the frequency and the diode parasitics get larger. In our simulation, the Avago HSMS285x diode with much higher parasitics exhibits more discrepancy between simulated and analytical optimal loads at 2.4 GHz than 400 MHz and 900 MHz. However, the discrepancy is still negligible. This means the frequency impact is negligible at least below 2.4 GHz.

The proposed numerical and closed-form methods have low computational complexity, which can provide a head start when designing a rectifier system. It provides very good accuracy without the need for either harmonic balance or transient simulation provided that the output voltage ripple is eliminated. Moreover, the proposed methods are also valid for general signals, for example, novel input signals such as the multisine waveform, with which the problem can quickly become infeasibly large for harmonic balance as the number of tones increases [7]. Another possible application is adaptive load control in an actual rectifier to ensure optimal efficiency. Implementation of such a control scheme constitute future work.

## Figures and Tables

**Table 1:** Parameters of two types of Schottky diode that are considered in the simulation.

	$i_{s}$	$R_{S}$	*N*	$C_{J0}$	*M*	$V_{J}$	$L_{S}$	$C_{P}$
SMS7630	5 μA	20 Ω	1.05	0.14 pF	0.4	0.51 V	0.05 nH	0.005 pF
HSMS285x	3 μA	25 Ω	1.06	0.18 pF	0.5	0.35 V	2 nH	0.08 pF

**Table 2:** Load resistance values used in the measurement.

	**R1**	**R2**	**R3**	**R4**	**R5**	**R6**	**R7** / **R8**	**R7**
Value [Ω]	16.2	100.3	328	558	822	1.2 k	1.99 k	3.29 k
	**R8**	**R9**	**R11 / R14**	**R10**	**R11**	**R12**	**R13**	**R14**
Value [Ω]	5.1 k	7.49 k	9.63 k	11.97 k	14.96 k	18.01 k	22 k	26 k

## Data Availability

The data can be made available upon request to the correspondence author.

## References

[1] Zhang Z., Pang H., Georgiadis A., Cecati C. (2018). Wireless power transfer—An overview. IEEE Trans. Ind. Electron..

[2] Li S., Mi C.C. (2014). Wireless power transfer for electric vehicle applications. IEEE J. Emerg. Sel. Top. Power Electron..

[3] Clerckx B., Costanzo A., Georgiadis A., Carvalho N.B. (2018). Toward 1G mobile power networks: RF, signal, and system designs to make smart objects autonomous. IEEE Microw. Mag..

[4] Ouda M.H., Mitcheson P., Clerckx B. (2018). Optimal operation of multitone waveforms in low RF-power receivers. Proceedings of the 2018 IEEE Wireless Power Transfer Conference (WPTC).

[5] Boaventura A., Collado A., Carvalho N.B., Georgiadis A. (2013). Optimum behavior: Wireless power transmission system design through behavioral models and efficient synthesis techniques. IEEE Microw. Mag..

[6] Pflug H.W., Keyrouz S., Visser H.J. (2016). Far-field energy harvesting rectifier analysis. Proceedings of the 2016 IEEE Wireless Power Transfer Conference (WPTC).

[7] Maas S.A. (2003). Nonlinear Microwave and RF Circuits.

[8] McSpadden J.O., Fan L., Chang K. (1998). Design and experiments of a high-conversion-efficiency 5.8-GHz rectenna. IEEE Trans. Microw. Theory Tech..

[9] Gao S.P., Zhang H., Ngo T., Guo Y. (2020). Lookup-table-based automated rectifier synthesis. IEEE Trans. Microw. Theory Tech..

[10] Yoo T.W., Chang K. (1992). Theoretical and experimental development of 10 and 35 GHz rectennas. IEEE Trans. Microw. Theory Tech..

[11] Guo J., Zhang H., Zhu X. (2014). Theoretical analysis of RF-DC conversion efficiency for class-F rectifiers. IEEE Trans. Microw. Theory Tech..

[12] Gu X., Guo L., Hemour S., Wu K. (2020). Optimum temperatures for enhanced power conversion efficiency (PCE) of zero-bias diode-based rectifiers. IEEE Trans. Microw. Theory Tech..

[13] Gu X., Hemour S., Wu K. (2020). Wireless Powered Sensors for Battery-Free IoT Through Multi-Stage Rectifier. Proceedings of the 2020 XXXIIIrd General Assembly and Scientific Symposium of the International Union of Radio Science.

[14] Bolos F., Blanco J., Collado A., Georgiadis A. (2016). RF energy harvesting from multi-tone and digitally modulated signals. IEEE Trans. Microw. Theory Tech..

[15] Hemour S., Zhao Y., Lorenz C.H.P., Houssameddine D., Gui Y., Hu C.M., Wu K. (2014). Towards low-power high-efficiency RF and microwave energy harvesting. IEEE Trans. Microw. Theory Tech..

[16] Lorenz C.H.P., Hemour S., Wu K. (2016). Physical mechanism and theoretical foundation of ambient RF power harvesting using zero-bias diodes. IEEE Trans. Microw. Theory Tech..

[17] Boaventura A.S., Carvalho N.B. (2011). Maximizing DC power in energy harvesting circuits using multisine excitation. Proceedings of the 2011 IEEE MTT-S International Microwave Symposium.

[18] Clerckx B., Bayguzina E. (2016). Waveform design for wireless power transfer. IEEE Trans. Signal Process..

[19] Moghadam M.R.V., Zeng Y., Zhang R. (2017). Waveform optimization for radio-frequency wireless power transfer. Proceedings of the 2017 IEEE 18th International Workshop on Signal Processing Advances in Wireless Communications (SPAWC).

[20] Morsi R., Jamali V., Ng D.W.K., Schober R. (2018). On the capacity of SWIPT systems with a nonlinear energy harvesting circuit. Proceedings of the 2018 IEEE International Conference on Communications (ICC).

[21] Kim K.W., Lee H.S., Lee J.W. (2018). Waveform design for fair wireless power transfer with multiple energy harvesting devices. IEEE J. Sel. Areas Commun..

[22] Varasteh M., Rassouli B., Clerckx B. (2017). Wireless information and power transfer over an AWGN channel: Nonlinearity and asymmetric Gaussian signaling. Proceedings of the 2017 IEEE Information Theory Workshop (ITW).

[23] Zawawi Z.B., Huang Y., Clerckx B. (2018). Multiuser wirelessly powered backscatter communications: Nonlinearity, waveform design, and SINR-energy tradeoff. IEEE Trans. Wirel. Commun..

[24] Yao L., Dolmans G., Romme J. (2021). On the Analytical Optimal Load Resistance of RF Energy Rectifier. Proceedings of the 2021 IEEE Wireless Power Transfer Conference (WPTC).

[25] SMS7630-040LF: 0402 Surface Mount Zero Bias Detector Schottky Diode. https://datasheet.octopart.com/SMS7630-040LF-Skyworks-Solutions-datasheet-8832283.pdf.

[26] Banwell T.C. (2000). Bipolar transistor circuit analysis using the Lambert W-function. IEEE Trans. Circuits Syst. Fundam. Theory Appl..

[27] De Vita G., Iannaccone G. (2005). Design criteria for the RF section of UHF and microwave passive RFID transponders. IEEE Trans. Microw. Theory Tech..

[28] Cunningham W.J. (1958). Introduction to Nonlinear Analysis.

[29] Le Polozec X. (2015). Input Impedance of Series Schottky Diode Detector at Low and High Power. https://www.researchgate.net/publication/277323751_Input_Impedance_of_Series_Schottky_Diode_Detector_at_Low_and_High_Power.

[30] Simscape: Model and Simulate Multi Domain Physical Systems. http://www.mathworks.com/products/simscape.

